# HIV and Sexually Transmitted Infection Testing Among Substance-Using Sexual and Gender Minority Adolescents and Young Adults: Baseline Survey of a Randomized Controlled Trial

**DOI:** 10.2196/30944

**Published:** 2022-07-01

**Authors:** Jayelin N Parker, Seul Ki Choi, Jose A Bauermeister, Erin E Bonar, Adam W Carrico, Rob Stephenson

**Affiliations:** 1 Center for Sexuality and Health Disparities University of Michigan Ann Arbor, MI United States; 2 Program for Sexuality, Technology, and Action Research University of Pennsylvania Philadelphia, PA United States; 3 Addiction Center, Department of Psychiatry University of Michigan Ann Arbor, MI United States; 4 School of Public Health University of Miami Miami, FL United States; 5 Department of Systems, Populations and Leadership School of Nursing University of Michigan Ann Arbor, MI United States

**Keywords:** testing, substance use, sexual minority, social determinants

## Abstract

**Background:**

Gay, bisexual, and other men who have sex with men and transgender individuals are more heavily affected by HIV and other sexually transmitted infections (STIs) than their cisgender, heterosexual peers. In addition, sexual and gender minorities who use substances are often at a further increased risk of HIV and other STIs. Increasing testing for HIV and other STIs allows this hardly reached population to receive early intervention, prevention, and education.

**Objective:**

We explored HIV and STI testing patterns among 414 sexual and gender minority adolescents and young adults aged 15 to 29 years who self-reported substance use and lived in southeastern Michigan.

**Methods:**

We analyzed data from the baseline survey of a 4-arm randomized controlled trial that aimed to examine the efficacy of a brief substance use intervention for creating gains in engagement in HIV prevention. We fit multinomial logistic regression models to 2 categorical HIV and STI testing variables (lifetime and previous 12 months) based on self-reports of testing (never, STIs only, HIV only, or both). In addition, we compared HIV and STI testing behaviors across demographic characteristics, structural factors, psychosocial barriers, substance use, and sexual behaviors.

**Results:**

Our findings showed that 35.5% (147/414) of adolescents and young adults reported not being tested for either HIV or STIs in the previous year, and less than half (168/414, 40.6%) of the sample achieved the Centers for Disease Control and Prevention recommendation of HIV and STI testing once per year. We observed HIV and STI testing disparities across sociodemographic (eg, sexual identity, education, and income) and health (eg, substance use) correlates. Specifically, cisgender gay men who have sex with men were more likely to report being tested for HIV compared with bisexual men and transgender individuals, who were more likely to be tested for STIs.

**Conclusions:**

This study illustrates the results of an HIV prevention intervention in southeastern Michigan showing the need for HIV prevention interventions that leverage structural factors, psychosocial barriers, and substance use as key drivers to achieve HIV and STI testing rates to meet the Centers for Disease Control and Prevention guidelines.

**Trial Registration:**

ClinicalTrials.gov NCT02945436; http://clinicaltrials.gov/ct2/show/NCT02945436

**International Registered Report Identifier (IRRID):**

RR2-10.2196/resprot.9414

## Introduction

### Background

In the United States, sexual and gender minority populations continue to experience disproportionate burdens of HIV and other sexually transmitted infections (STIs) compared with heterosexual and cisgender populations. Although <0.5% of the adult American population lives with HIV [1], it is estimated that the prevalence of HIV is significantly higher among sexual and gender minorities—14.1% of transgender women and 3.2% of transgender men live with HIV [1], and 16.6% of gay, bisexual, and other men who have sex with men (GBMSM) live with HIV [2]. In 2018, 69% of new HIV diagnoses were among GBMSM [3] and, despite efforts to reduce HIV, the incidence rates among GBMSM aged <30 years continue to increase [4]. Although transgender individuals comprise only 2% of new HIV diagnoses, in 2018, the largest percentage (27%) of diagnoses of HIV infection among transgender persons was for transgender female adults and adolescents aged 25 to 29 years, followed by transgender female adults and adolescents aged 20 to 24 years (25%) [5]. A 2019 systematic review and meta-analysis found that an estimated 14% of transgender women live with HIV, with strong disparities by race and ethnicity—an estimated 44% of Black or African American transgender women, 26% of Hispanic and Latina transgender women, and 7% of White transgender women live with HIV [6].

GBMSM and transgender populations have also been shown to be at an increased risk of STIs other than HIV [7-10]. Although women in the United States have a higher disease burden of chlamydia than men, from 2014 to 2018, there was an increase of 37.8% in STI infection rates among men, a rise primarily credited to increasing incident STI rates among GBMSM in particular [11]. Across 18 STI clinics within 9 US STI surveillance regions, the median site-specific chlamydia prevalence for GBMSM was 5.8% in urethral cases, 16.1% in rectal cases, and 2.7% in pharyngeal cases in 2018 [11]. A recent systematic review found prevalence ranges as follows among transgender women: syphilis (1.4%-50.4%), gonorrhea (2.1%-19.1%), and chlamydia (2.7%-24.7%) [12].

Within sexual and gender minority populations, the rates of HIV and STIs are significantly higher among substance using individuals [13,14]. In a recent cross-sectional sample of 2216 youth (aged 12-26 years) living with HIV, 32.9% of participants reported weekly or more frequent tobacco use, 27.5% reported marijuana use, 21.3% reported alcohol use, and 22.5% reported other illicit drug use [15]. Concurrently addressing substance use and HIV and STI prevention and treatment has the potential to create significant gains in reducing rates of HIV and STIs among sexual and gender minority communities [13,16]. Although there is an abundance of literature demonstrating the high rates of HIV among young GBMSM [17-21], young transgender individuals [16,22,23], and people who use or misuse substances [24-26], little research attention has focused on the intersection of these marginalized groups and has sought to understand the patterns of HIV and STI testing among young, substance using, sexual and gender minority individuals.

Testing for HIV and other STIs is a critical entry point into the HIV and STI care continuums, allowing people to be aware of their status, obtain treatment, and take appropriate action to prevent further transmission [27]; however, testing rates are often low among young, substance using, sexual and gender minority communities [28-33]. A recent study with transgender individuals showed that, of those at risk of contracting HIV, 23% had never been tested for HIV [34]. In addition, there are wide variations in testing within sexual and gender minority populations by race and ethnicity—for example, Latinx GBMSM are tested less frequently than other GBMSM [35]. In a recent study of testing behaviors among GBMSM, 8% of HIV tests delivered were to first-time testers, and 70.7% of first-time testers were among racial and ethnic minorities [36]. The low rates of HIV and STI testing among sexual and gender minorities have been attributed to anticipated stigma, fear, and perception of individual risk, which often act as barriers to willingness and ability to be tested for HIV and STIs [37-41]. In addition, structural barriers such as anticipated or previous poor interaction with or trust in medical providers and stigma often prevent young, sexual and gender minority individuals from seeking testing for HIV and other STIs [39,42,43].

This study focused on understanding the factors associated with HIV and STI testing among sexual and gender minority adolescents and young adults (AYA) in Detroit, Michigan. The Detroit Metro Area contains the most heavily concentrated group of people living with HIV in the state of Michigan, where the highest HIV and STI infection rates are among those living in the city of Detroit [44]. In 2018, in the Detroit Metro Area, 75.5% of people living with HIV were people of color, 55% were GBMSM, and 18.3% were between the ages of 13 and 29 years [45]. Young African American or Black GBMSM (aged 15-19 years) were the only group of people to experience an increase in new HIV infections over the last 2 decades in Michigan, where only 0.1% of the population are young GBMSM [45]. The transgender community accounted for 1.8% of people living with HIV in Detroit, where 82.6% of transgender individuals living with HIV are Black or African American [45]. GBMSM account for 22% of new HIV infections [45] such that, in 2018, young GBMSM were 270 times more likely to be diagnosed with HIV than young non-GBMSM in the Detroit Metro Area [45].

### Objectives

This study used baseline data from a randomized controlled trial with substance using, sexual and gender minority AYA recruited in southeastern Michigan to understand patterns of testing for HIV and STIs. The primary aim of this analysis was to understand HIV and STI testing behaviors among substance using sexual and gender minority AYA, a group for whom it is critical to develop interventions that can increase testing uptake. Understanding the social determinants (eg, gender identity, sexual identity, and education) associated with HIV and STI testing among this vulnerable population has the potential to inform the development of interventions tailored to the unique needs of a population experiencing substance use and multiple structural and interpersonal barriers to engaging in routine testing.

## Methods

### Study Procedures and Participants

We analyzed data from the baseline survey of a 4-arm randomized controlled trial of substance using, sexual and gender minority AYA (aged 15-29 years; N=414) that examined the efficacy of a brief substance use intervention for creating gains in engagement in HIV prevention. The eligibility criteria were as follows: being aged 15 to 29 years at the time of screening; living in southeastern Michigan (based on eligible zip codes); identifying as a man, male, or transgender person; having had condomless oral or anal sex at least once in the 6 months before screening; and having had at least one binge drinking or substance misuse experience (≥5 standard alcoholic beverages in a single setting) in the last 3 months before screening. The study recruitment period was from April 2017 to August 2019. Participants were walked through consent by a staff member, ensuring that they could opt out of the study at any time and were not forced to participate.

### Ethics Approval

The study was approved by the Institutional Review Board of the University of Michigan (HUM00105125). The trial is also registered at ClinicalTrials.gov (NCT02945436). More information regarding the randomized controlled trial is available in a detailed protocol paper [46].

### Measures

#### HIV and STI Testing

The participants responded whether they had ever been tested for HIV or STIs in their lifetime. On the basis of the participants’ responses, we created a categorical variable of lifetime HIV and STI testing with 4 groups: never tested for either HIV or STIs, tested for STIs only, tested for HIV only, or tested for both HIV and STIs. If they had been tested in the past, the participants responded whether they had been tested in the 12 months before the survey. Similar to lifetime testing, we created a categorical variable of previous–12-month HIV and STI testing: never tested for HIV and STIs, tested for STIs only, tested for HIV only, or tested for both HIV and STIs.

#### Individual Factors

We included demographic characteristics (ie, age, race, ethnicity [Hispanic or Latinx], gender identity, sexual identity, and disability) in the analysis. Age was dichotomized as 15 to 21 years or 22 to 29 years. The participants were asked about their gender identity using 4 response options (cisgender, transgender men, transgender women, or nonbinary). Owing to the small number of responses in each category, transgender men, transgender women, and nonbinary individuals were combined into *other*, creating a dichotomized variable for gender identity (cisgender or other). Sexual identity was categorized as gay, bisexual, and other.

#### Structural Factors

The participants self-reported incarceration history (never incarcerated, incarcerated in their lifetime but not incarcerated in the last 12 months, and incarcerated in the last 12 months), employment (full-time or other), health insurance enrollment (currently insured or not insured), and housing stability (residing in stable housing or not residing in stable housing). The participants responded with the highest level of education that they had completed (some high school, graduated high school or obtained General Educational Development, and some college and higher). The participants reported their household income in the previous year from all sources before tax, which we divided into <US $15,000, ≥US $15,000 but <US $40,000, and ≥US $40,000.

#### HIV-Related Characteristics

The participants indicated how likely they felt they were to contract HIV with responses on a 4-point Likert scale from *very likely* to *very unlikely*. The participants also responded how likely they felt they were to contract HIV compared with their peers in the next 10 years using the same response options. The participants reported their awareness of pre-exposure prophylaxis (PrEP; yes or no) and their use of PrEP (never used, past use, or current use). We merged these 2 variables and created a PrEP continuum variable with 3 response options (unaware or aware but never used, past use, and current use).

#### Mental Health

The 7-item General Anxiety Disorder scale assessed frequency of anxiety symptoms in the previous 2 weeks; symptomatology comprised (1) minimal, (2) mild, (3) moderate, and (4) severe [47]. Depressive symptoms in the previous week were assessed using a shortened version of the Center for Epidemiological Studies-Depression Scale [48]. The frequency of symptomatology comprised (1) never, (2) hardly ever, (3) some of the time, and (4) most of the time. A cutoff score of ≥16 indicates clinically relevant depressive symptomatology.

#### Substance Use

We used the Alcohol Use Disorders Identification Test [49], a 10-item screener to measure hazardous drinking in the previous 3 months, which is defined by a total score of ≥8. Items based on the Alcohol, Smoking, and Substance Involvement Screening Test [50] were used to characterize previous–3-month substance consumption among the participants. This resulted in the creation of dichotomous variables (yes or no) representing previous–3-month smoking, use of cannabis, stimulants (cocaine, cocaine powder, crack cocaine, or methamphetamine), sedatives (prescribed Xanax, Klonopin, or Valium), club drugs (ecstasy, ketamine, or gamma-hydroxybutyrate opioids), opioids (prescribed opioids or heroin), hallucinogens (mushrooms, lysergic acid diethylamide, or phencyclidine), and amyl-nitrites (poppers or rush). Given the low frequency of drugs used other than cannabis (and tobacco), we created two composite drug use variables with the remaining 6 individual drugs assessed: (1) other drug use (yes or no) and (2) the number of other drugs used in the previous 3 months (continuous, range 0-6).

#### Sexual Risk Behaviors

The participants reported the total incidence of receptive or insertive condomless anal intercourse in the previous 3 months. In addition, the participants reported the total incidence of condomless vaginal intercourse in the previous 3 months.

### Statistical Analysis

Descriptive analyses were conducted to characterize the sample (eg, means, SDs, and proportions). Bivariate multinomial logistic regression models were fitted to the 2 categorical outcomes measuring lifetime and previous–12-month HIV and STI testing. Among the 4 different HIV and STI testing outcomes (never, STIs only, HIV only, or both), the largest group was selected as the reference group. Each model compared HIV and STI testing categories according to demographic characteristics, structural factors, psychosocial barriers, and substance use and sexual behaviors. Dummy variables were created for gender and sexual identity, and ordinal variables (education, income, incarceration, HIV likely, PrEP awareness and use, and anxiety) were considered continuous variables in the modeling. Models were fit using SAS statistical software (version 9.4; SAS Institute Inc).

## Results

### Sample Description

Multimedia Appendix 1 summarizes the descriptive information of the participants in detail. Briefly, the mean age of the participants was 22.5 (SD 3.22) years. More than half identified as White (285/414, 68.8%), cisgender male (331/414, 80%), and gay (270/414, 65.2%). Most had secure housing (261/414, 63%) and health insurance (347/414, 83.8%) and had completed at least some college (295/414, 71.3%).

Regarding *lifetime* testing, 62.6% (259/414) had been tested for both HIV and STIs, 7% (29/414) had been tested only for STIs, 10.4% (43/414) had been tested only for HIV, and 20% (83/414) had never been tested for either. In the previous *12 months*, 40.6% (168/414) had been tested for both HIV and STIs, 15.5% (64/414) had been tested only for HIV, 8.5% (35/414) had been tested only for STIs, and 35.5% (147/414) had not been tested for either HIV or STIs. One-fourth of the participants (110/414, 26.6%) reported a lifetime diagnosis of an STI (49/414, 11.8%) in the previous *12 months*. Most participants (355/414, 85.7%) were either unaware of PrEP or had not used it in the past.

Approximately half of the participants reported moderate to severe anxiety symptoms (175/414, 42.3%) and depressive symptoms (262/414, 63.3%). Most participants (284/414, 68.6%) reported experience with cannabis, and nearly half of the participants (178/414, 43%) reported experience with other drugs in the previous 3 months. More than half of the participants (257/414, 62.1%) reported condomless anal intercourse, and 14% (58/414) had had condomless vaginal intercourse in the previous 3 months.

### Associations With HIV and STI Testing

#### Overview

Multimedia Appendices 2 and 3 show sample characteristics across HIV and STI testing in their lifetime and in the previous 12 months, respectively. Multimedia Appendices 4 and 5 show the results of the multinomial regression models demonstrating the relationships between independent variables and HIV and STI testing (lifetime and previous 12 months, respectively).

#### Demographic Characteristics

Older participants were more likely to have been tested for both HIV and STIs in their lifetime (odds ratio [OR] 1.27, 95% CI 1.16-1.38) and in the previous 12 months (OR 1.12, 95% CI 1.04-1.21). In addition, older participants were less likely to have only been tested for STIs in their lifetime (OR 0.82, 95% CI 0.72-0.94), whereas older participants were more likely to have been tested for HIV in the previous 12 months (OR 1.12, 95% CI 1.02-1.23). Participants who identified as cisgender were 77% less likely to have been tested only for STIs (OR 0.23, 95% CI 0.10-0.50) than to have been tested for both HIV and STIs in their lifetime, whereas cisgender participants were 5 times more likely to have only been tested for HIV (OR 5.16, 95% CI 1.53-17.45) in the previous 12 months. Participants who identified as gay were less likely to have been tested only for STIs in their lifetime (OR 0.29, 95% CI 0.13-0.64) and in the previous 12 months (OR 0.45, 95% CI 0.21-0.94) than those who had been tested for both HIV and STIs. However, participants who identified as bisexual were more likely to have been tested only for STIs in their lifetime (OR 4.24, 95% CI 1.87-9.59) and to have not been tested for either HIV or STIs in the previous 12 months (OR 1.97, 95% CI 1.10-3.52). Participants with higher levels of education were more likely to have been tested for HIV and STIs in their lifetime (OR 2.19, 95% CI 1.48-3.25) and in the previous 12 months (OR 1.76, 95% CI 1.19-2.56) than to have never been tested for either HIV or STIs. Those with higher incomes were less likely to have only been tested for STIs in their lifetime (OR 0.52, 95% CI 0.28-0.96) than to have been tested for both HIV and STIs.

#### HIV-Related Characteristics

Participants who reported higher awareness and use of PrEP were less likely to have been tested only for HIV in their lifetime (OR 0.25, 95% CI 0.07-0.83) and in the previous 12 months (OR 0.36, 95% CI 0.19-0.70).

#### Psychosocial Barriers

Participants with greater anxiety symptoms were more likely to have only been tested for STIs in their lifetime (OR 1.42, 95% CI 1.01-1.99), although depressive symptomatology was not associated with HIV and STI testing.

#### Substance Use Behaviors

Tobacco use and hazardous drinking were not associated with HIV and STI testing in their lifetime and in the previous 12 months; however, sedative and opioid use were associated with HIV and STI testing in their lifetime, and cannabis, opioid, and amyl-nitrite use was associated with previous-year HIV and STI testing. Participants who used sedatives (OR 2.05, 95% CI 1.04-4.03) and opioids (OR 4.38, 95% CI 1.58-12.16) were more likely to not have been tested for either HIV or STIs in their lifetime compared with those who had been tested for both. AYA who used opioids were more likely to have not been tested for HIV or STIs in the previous 12 months (OR 3.64, 95% CI 1.15-11.56), whereas AYA who used amyl-nitrites were less likely to have been tested for either HIV or STIs in the previous 12 months (OR 0.52, 95% CI 0.27-0.97). Participants who used cannabis were less likely to have only been tested for HIV (OR 0.54, 95% CI 0.29-0.98) in the previous 12 months.

#### Sexual Behaviors

Participants who reported condomless anal intercourse were more likely to have been tested for both HIV and STIs in their lifetime (OR 2.89, 95% CI 1.74-4.79) and in the previous 12 months (OR 2.76, 95% CI 1.73-4.40) compared with those who had not been tested for either HIV or STIs. Specifically, receptive condomless anal intercourse was associated with HIV and STI testing in their lifetime (OR 0.50, 95% CI 0.30-0.82) and in the previous 12 months (OR 0.46, 95% CI 0.29-0.72). Similarly, participants who reported condomless anal intercourse (OR 0.26, 95% CI 0.12-0.58), especially receptive condomless anal intercourse (OR 0.27, 95% CI 0.12-0.64), in the previous 3 months were less likely to have been tested only for STIs in their lifetime. However, participants who reported condomless vaginal intercourse (OR 3.19, 95% CI 1.34-7.62), especially receptive condomless vaginal intercourse (OR 3.30, 95% CI 1.20-9.07), were more likely to have been tested for only an STI compared with testing for both HIV and STIs in their lifetime.

## Discussion

### Principal Findings

In this sample of substance using sexual and gender minority AYA, approximately two-thirds of the participants reported being tested for both HIV and STIs in their lifetime (259/414, 62.6%); however, 20% (83/414) of the sample had not been tested for either in the previous year, a concerning rate given the Centers for Disease Control and Prevention (CDC) recommendation that all sexually active, young, substance using, sexual and gender minority individuals should be tested for STIs annually and for HIV every 3 to 6 months [51]. Our study findings show that 40.6% (168/414) of the sample followed the recommendation of annual testing for both HIV and STIs, which differs from previous studies—national HIV testing rates for young men who have sex with men varied from 15% to 30% [52,53], 15% of transgender youth aged 15 to 24 years were tested for HIV and 23% were tested for STIs in the previous year [54], and 87% of Black sexual minority men aged 18 to 30 years were tested for HIV in the previous year [55]. Our sample showed greater adherence to testing behaviors than previous studies, although 59.5% (246/414) of the sample did not follow the CDC’s recommendations for routine HIV and STI testing. Previous studies have illustrated that young GBMSM report a lack of awareness of the need for HIV testing, fear of results and rejection, physical and economic access issues, testing-related stigma, and unfriendly testing environments [56]. Participants with higher levels of PrEP awareness and use were more likely to engage in HIV and STI testing, likely a function of the need for routine testing for PrEP users. Consistent with previous studies [57,58], participants with lower educational attainment and income were less likely to engage in HIV and STI testing, pointing to a lack of social and economic capital as a significant barrier to engaging in testing. However, each of these barriers to testing is malleable, and interventions that work directly to eliminate barriers to HIV and STI testing and promote knowledge of its benefits should be considered a research and programmatic priority for substance using sexual and gender minority AYA [59-61].

Transgender individuals and bisexual men reported having been tested for STIs but not HIV, whereas cisgender gay men were more likely to have been tested for HIV than for STIs. These patterns of HIV and STI testing may be attributed to differing perceived susceptibility to HIV or STIs across sexual and gender minority communities but also to the targeting that is often used in HIV and STI programs. HIV testing has long focused on the need for GBMSM to be tested regularly, and only recently in the epidemic have promotional materials begun to include the transgender community [62,63]. However, STI programs have focused on the general population, in particular adolescents, and it is possible that bisexual and transgender populations are receiving more messaging about the need for STI testing or resonate more clearly with the material used to promote STI testing, if any. Similarly, those who reported condomless vaginal intercourse were more likely to have been tested only for STIs, and those reporting condomless anal intercourse were less likely to have been tested for HIV and STIs compared with those who had been tested for both HIV and STIs. Just as programs may be targeting testing differentially by demographic characteristics, they may be targeting their programs based on risk, targeting HIV and STI testing more toward GBMSM, who are more likely to engage in condomless anal intercourse. This result indicates the need for HIV and STI programs to think critically about how they promote testing and to ensure the inclusion of all sexual and gender minority AYA and their unique prevention needs in their promotional efforts.

Both self-reported mental health and substance use were associated with HIV and STI testing. Participants with higher levels of anxiety were more likely to have only been tested for STIs, perhaps suggesting that anxiety is a barrier to engaging in HIV testing, although further research is warranted to understand the pathways between anxiety and engagement in testing. Opioid- and sedative using participants were more likely to not have been tested for either HIV or STIs compared with those who had been tested for both HIV and STIs in their lifetime. In general, substance using AYA may have specific barriers to engaging in testing—for example, fear of having to report their substance use, which prevents them from seeking testing services [4], whereas other samples of substance using AYA report barriers such as not feeling at risk or not being offered a test [64]. However, it is important to note that patterns could vary based on misuse versus medical use, which was not distinguished in our sample and is a direction for future research. However, in this sample, inhalant using participants were more likely to have been tested for both HIV and STIs in the previous year. Previous studies have demonstrated associations between amyl-nitrate use and risky sexual behavior, and it is plausible that these participants engage in testing in recognition of their risk-taking behaviors [65], especially when amyl-nitrites are almost exclusively used to facilitate anal intercourse [66]. The results indicate that there is no single relationship between substance use and engagement in HIV and STI testing and that it is important to look critically at the relationships between individual types of substance use (ie, opioids vs cannabis vs amyl-nitrites) when attempting to design programs that promote testing for substance using AYA.

### Limitations

There are several limitations to this analysis. First, the cross-sectional nature of the data precludes any inference of causality, but it does provide a foundation for further examinations to draw from. Second, the small number of participants reporting noncisgender identities precluded a thorough examination of associations between gender identity and HIV and STI testing, a common limitation of the literature, which often aggregates gender identities [67-69]. Third, participants may be susceptible to demand characteristics or underreporting of sensitive behaviors, although this limitation is tempered by the use of confidentiality assurances and computerized self-report, which enhances the validity of self-report for substance use (which is reliable and valid) [70] and sexual risk behaviors, and there is a wealth of previous studies that have shown the validity of self-reported measures of substance use [71,72]. Finally, this trial recruited substance using GBMSM; however, there were 23 participants who reported discrepant responses in substance use and drinking between the screener and baseline survey. Of these 23 participants, 15 (65%) reported drinking but not binge drinking, and 8 (35%) reported drug use at some point in their lives but not in the previous 3 months.

### Conclusions

The goal of this study was to understand the HIV and STI testing behaviors of substance using sexual and gender minority AYA living in southeastern Michigan. Our results show that, contrary to CDC testing guidelines, 35.5% (147/414) of AYA had not been tested for either HIV or STIs in the previous year, and less than half (168/414, 40.6%) meet the CDC testing recommendations. To meet the CDC’s guidelines on testing, HIV and STI testing interventions need to recognize the specific barriers to engaging in testing experienced by substance using sexual and gender minority AYA, who experience multiple layers of potential stigma grounded in their age, sexual and gender identities, and substance use behavior. Central to this is the recognition that not all substances are linked to testing behaviors in the same way, and research and programmatic efforts need to consider the differential testing needs, attitudes, and barriers of different types of substance using AYA.

## Appendix

Multimedia Appendix 1 Demographic and behavioral characteristics of a sample of substance-using sexual and gender minority adolescents and young adults in the Detroit Metro Area (aged 15-29 years; N=414).

Multimedia Appendix 2 Distribution of demographic characteristics, structural factors, psychosocial barriers, and substance use and sexual behaviors by lifetime HIV testing among substance-using sexual and gender minority adolescents and young adults (N=414).

Multimedia Appendix 3 Distribution of demographic characteristics, structural factors, psychosocial barriers, and substance use and sexual behaviors by previous-year HIV and sexually transmitted infection testing among substance-using sexual and gender minority adolescents and young adults (N=414).

Multimedia Appendix 4 Odds of lifetime HIV and sexually transmitted infection testing by demographic characteristics, structural factors, psychosocial barriers, and substance use and sexual behaviors among substance-using sexual and gender minority adolescents and young adults (N=414).

Multimedia Appendix 5 Odds of previous-year HIV and sexually transmitted infection testing by demographic characteristics, structural factors, psychosocial barriers, and substance use and sexual behaviors among substance-using sexual and gender minority adolescents and young adults (N=414).

## Abbreviations

AYA: adolescents and young adults
CDC: Centers for Disease Control and Prevention
GBMSM: gay, bisexual, and other men who have sex with men
OR: odds ratio
PrEP: pre-exposure prophylaxis
STI: sexually transmitted infection
