# Supplementary material for: HIV and Sexually Transmitted Infection Testing Among Substance-Using Sexual and Gender Minority Adolescents and Young Adults: Baseline Survey of a Randomized Controlled Trial
Source: JMIR Public Health Surveill. 2022 Jul 1;8(7):e30944. doi: 10.2196/30944 (PMC9288102; doi:10.2196/30944)
Supplement: Multimedia Appendix 1 [file publichealth_v8i7e30944_app1.pdf]

Multimedia Appendix 1. Demographic and behavioral characteristics of a sample of substance-using sexual and gender minority adolescents and young adults in the Detroit Metro Area (aged 15-29 years; N=414).

Characteristic	Values
**Demographics**	
**Age (years), mean (SD)**	22.5 (3.2)
15 to 21, n (%)	160 (38.6)
22 to 29, n (%)	254 (61.4)
**Ethnicity, n (%)**	
Hispanic	40 (9.7)
Non-Hispanic	374 (90.3)
**Race, n (%)**	
White	285 (68.8)
Black or African American	56 (13.5)
Multiracial	37 (8.9)
Other	36 (8.7)
**Gender identity, n (%)**	
Cisgender man	331 (80)
**Binary transgender**	59 (14.3)
Male to female	13 (3.1)
Female to male	46 (11.1)
Nonbinary	24 (5.8)
**Sexual identity, n (%)**	
Gay	270 (65.2)
Bisexual	77 (18.6)
Other	67 (16.2)
**Education, n (%)**	
Some high school	26 (6.3)

		High school graduate/GED[a]	93 (22.5)
		Some college or higher	295 (71.3)
	**Employment, n (%)**		
		Employed full-time	157 (37.9)
		Other	257 (62.1)
	**Housing, n (%)**		
		Stable or permanent	261 (63)
		Temporary, unstable, homeless, or other	153 (37)
	**Annual income (US $), n (%)**		
		≤14,999	157 (37.9)
		15,000 to 39,999	129 (31.2)
		≥40,000	82 (19.8)
	**Disability, n (%)**		
		Yes	68 (16.4)
		No	345 (83.3)
	**Health insurance—current, n (%)**		
		Yes	347 (83.8)
		No	67 (16.2)
	**Incarceration, n (%)**		
		Never	329 (79.5)
		Incarcerated in their lifetime but not incarcerated in the last 12 months	65 (15.7)
		Incarcerated in the last 12 months	20 (4.8)
**HIV and STI-related characteristics, n (%)**			
	**HIV AND STI[b] testing, lifetime**		
		None	83 (20)
		STIs only	29 (7)

	HIV only	43 (10.4)
	Both	259 (62.6)
**Lifetime STI diagnosis**		
	Yes	110 (26.6)
	No	304 (73.4)
**HIV and STI testing, previous year**		
	None	147 (35.5)
	STIs only	35 (8.5)
	HIV only	64 (15.5)
	Both	168 (40.6)
**Previous-year STI diagnosis**		
	Yes	49 (11.8)
	No	365 (88.2)
**Likelihood of HIV infection in the future**		
	Very likely	3 (0.7)
	Somewhat likely	40 (9.7)
	Somewhat unlikely	170 (41.1)
	Very unlikely	201 (48.6)
**Likelihood of HIV infection in the next 10 years compared with peers**		
	Very likely	17 (4.1)
	Somewhat likely	74 (17.9)
	Somewhat unlikely	161 (38.9)
	Very unlikely	162 (39.1)
**PrEP[c] continuum**		
	Unaware/aware	355 (85.7)
	Past use	26 (6.3)

Current use	33 (8)
**Mental health, n (%)**	
**Anxiety—last 2 weeks**	
Minimal	120 (29)
Mild	119 (28.7)
Moderate	77 (18.6)
Severe	98 (23.7)
**Depression symptoms—previous week**	
Yes	262 (63.3)
No	160 (38.6)
**Substance use—previous 3 months**	
**Tobacco use, n (%)**	
Yes	338 (81.6)
No	76 (18.4)
**Hazardous drinking, n (%)**	
Yes	147 (35.5)
No	267 (64.5)
Cannabis use, n (%)	284 (68.6)
**Other drug use, n (%)**	178 (43)
Drugs used among "other drug use," mean (SD)	0.79 (1.23)
Stimulants, n (%)	98 (23.7)
Sedatives, n (%)	50 (12.1)
Club drugs, n (%)	36 (8.7)
Opioids, n (%)	20 (4.8)
Hallucinogens, n (%)	58 (14)
Amyl-nitrites, n (%)	65 (15.7)

**Sexual risk behavior—previous 3 months, n (%)**			
	**CAI**[d]		257 (62.1)
		Receptive CAI	214 (51.7)
		Insertive CAI	163 (39.4)
	**CVI**[e]		58 (14)
		Receptive CVI	32 (7.7)
		Insertive CVI	28 (6.8)

[a]GED: General Educational Development.

[b]STI: sexually transmitted infection.

[c]PrEP: pre-exposure prophylaxis.

[d]CAI: condomless anal intercourse.

[e]CVI: condomless vaginal intercourse.
