# Supplementary material for: HIV and Sexually Transmitted Infection Testing Among Substance-Using Sexual and Gender Minority Adolescents and Young Adults: Baseline Survey of a Randomized Controlled Trial
Source: JMIR Public Health Surveill. 2022 Jul 1;8(7):e30944. doi: 10.2196/30944 (PMC9288102; doi:10.2196/30944)
Supplement: Multimedia Appendix 2 [file publichealth_v8i7e30944_app2.pdf]

Multimedia Appendix 2. Distribution of demographic characteristics, structural factors, psychosocial barriers, and substance use and sexual behaviors by lifetime HIV testing among substance-using sexual and gender minority adolescents and young adults (N=414).

Characteristic	None (n=83)	STIs[a] only (n=29)	HIV only (n=43)	Both (n=259)
**Demographics**				
Age (years), mean (SD)	20.8 (2.87)	21.2 (3.25)	23.4 (3.52)	23.0 (3.04)
**Ethnicity, n (%)**				
Hispanic	12 (14.5)	5 (17.2)	1 (2.3)	22 (8.5)
Non-Hispanic	71 (85.5)	24 (82.8)	42 (97.7)	237 (91.5)
**Race, n (%)**				
White	60 (72.3)	23 (79.3)	26 (60.5)	176 (68)
Black or African American	6 (7.2)	2 (6.9)	8 (18.6)	40 (15.4)
Multiracial	10 (12)	1 (3.4)	4 (9.3)	22 (8.5)
Other	7 (8.4)	3 (10.3)	5 (11.6)	21 (8.1)
**Gender identity, n (%)**				
Cisgender men	70 (84.3)	14 (48.3)	39 (90.7)	208 (80.3)
Transgender men	9 (10.8)	12 (41.4)	2 (4.7)	23 (8.9)
Transgender women	1 (1.2)	2 (6.9)	2 (4.7)	8 (3.1)
Nonbinary	3 (3.6)	1 (3.4)	0 (0)	20 (7.7)
**Sexual identity, n (%)**				
Gay	54 (65.1)	11 (37.9)	29 (67.4)	176 (68)
Bisexual	17 (20.5)	12 (41.4)	11 (25.6)	37 (14.3)
Other	12 (14.5)	6 (20.7)	3 (7)	46 (17.8)
**Education, n (%)**				
Some high school	11 (13.3)	3 (10.3)	1 (2.3)	11 (4.2)
High school graduate/GED[b]	24 (28.9)	9 (31)	15 (34.9)	45 (17.4)

	Some college or higher	48 (57.8)	17 (58.6)	27 (62.8)	203 (78.4)
**Employment, n (%)**					
	Employed full-time	25 (30.1)	9 (31)	22 (51.2)	101 (39)
	Other	58 (69.9)	20 (69)	21 (48.8)	158 (61)
**Housing, n (%)**					
	Stable or permanent	51 (61.4)	18 (62.1)	25 (58.1)	167 (64.5)
	Temporary, unstable, homeless, or other	32 (38.6)	11 (37.9)	18 (41.9)	92 (35.5)
**Yearly income (US $), n (%)**					
	~14,999	30 (36.1)	15 (51.7)	15 (34.8)	97 (37.4)
	15,000 to 39,999	21 (25.3)	10 (34.5)	12 (27.9)	86 (33.2)
	~40,000	19 (22.9)	1 (3.4)	11 (25.5)	51 (19.7)
**Disability, n (%)**					
	Yes	10 (12)	4 (13.8)	8 (18.6)	46 (17.8)
	No	73 (88)	25 (86.2)	35 (81.4)	212 (81.9)
**Health insurance—current, n (%)**					
	Yes	72 (86.7)	23 (79.3)	32 (74.4)	220 (84.9)
	No	11 (13.3)	6 (20.7)	11 (25.6)	39 (15.1)
**Incarceration, n (%)**					
	Never	65 (78.3)	23 (79.3)	32 (74.4)	209 (80.7)
	Incarcerated in their lifetime but not incarcerated in the last 12 months	14 (16.9)	4 (13.8)	5 (11.6)	42 (16.2)
	Incarcerated in the last 12 months	4 (4.8)	2 (6.9)	6 (14)	8 (3.1)
**HIV-related characteristics, n (%)**					
**Likelihood of HIV infection in the future**					
	Very likely	1 (1.2)	0 (0)	0 (0)	2 (0.8)
	Somewhat likely	9 (10.8)	0 (0)	2 (4.7)	29 (11.2)
	Somewhat unlikely	35 (42.2)	14 (48.3)	21 (48.8)	100 (38.6)

Very unlikely	38 (45.8)	15 (51.7)	20 (46.5)	128 (49.4)
**Likelihood of HIV infection in the next 10 years**				
Very likely	1 (1.2)	0 (0)	0 (0)	16 (6.2)
Somewhat likely	12 (14.5)	6 (20.7)	10 (23.2)	46 (17.8)
Somewhat unlikely	40 (48.2)	12 (41.4)	15 (34.9)	94 (36.3)
Very unlikely	30 (36.1)	11 (37.9)	18 (41.9)	103 (39.8)
**PrEP[c] continuum**				
Unaware/aware	83 (100)	28 (96.6)	41 (95.3)	203 (78.4)
Past use	0 (0)	0 (0)	2 (4.7)	24 (9.3)
Current use	0 (0)	1 (3.4)	0 (0)	32 (12.4)
**Mental health, n (%)**				
**Anxiety—last 2 weeks**				
Minimal	24 (28.9)	2 (6.9)	15 (34.9)	79 (30.5)
Mild	24 (28.9)	11 (37.9)	12 (27.9)	72 (27.8)
Moderate	16 (19.3)	7 (24.1)	6 (14)	48 (18.5)
Severe	19 (22.9)	9 (31)	10 (23.3)	60 (23.2)
**Depression symptoms—previous week**				
Yes	54 (65.1)	22 (75.9)	24 (55.8)	152 (58.7)
No	28 (33.7)	7 (24.1)	19 (44.2)	106 (40.9)
**Substance use—previous 3 months, n (%)**				
**Tobacco use**				
Yes	69 (83.1)	27 (93.1)	38 (88.4)	204 (78.8)
No	14 (16.9)	2 (6.9)	5 (11.6)	55 (21.2)
**Hazardous drinking**				
Yes	26 (31.3)	8 (27.6)	14 (32.6)	99 (38.2)
No	57 (68.7)	21 (72.4)	29 (67.4)	160 (61.8)

	Cannabis use		58 (69.9)	22 (75.9)	26 (60.5)	178 (68.7)
	**Other drug use**		36 (43.4)	12 (41.4)	17 (39.5)	113 (43.6)
		Stimulants	20 (24.1)	9 (31)	10 (23.3)	59 (22.8)
		Sedatives	16 (19.3)	2 (6.9)	5 (11.6)	27 (10.4)
		Club drugs	7 (8.4)	2 (6.9)	4 (9.3)	23 (8.9)
		Opioids	9 (10.8)	1 (3.4)	3 (7)	7 (2.7)
		Hallucinogens	17 (20.5)	5 (17.2)	5 (11.6)	31 (12)
		Amyl-nitrites	9 (10.8)	2 (6.9)	6 (14)	48 (18.5)
**Sexual risk behavior—previous 3 months, n (%)**						
	**CAI**[d]		37 (44.6)	11 (37.9)	28 (65.1)	181 (69.9)
		Receptive CAI	34 (41)	8 (27.6)	21 (48.8)	151 (58.3)
		Insertive CAI	18 (21.7)	8 (27.6)	18 (41.9)	119 (45.9)
	**CVI**[e]		12 (14.5)	9 (31)	5 (11.6)	32 (12.4)
		Receptive CVI	4 (4.8)	6 (20.7)	3 (7)	19 (7.3)
		Insertive CVI	8 (9.6)	3 (10.3)	2 (4.7)	15 (5.8)

[a]STIs: sexually transmitted infections.
[b]GED: General Educational Development.
[c]PrEP: pre-exposure prophylaxis.
[d]CAI: condomless anal intercourse.
[e]CVI: condomless vaginal intercourse.
