# Supplementary material for: HIV and Sexually Transmitted Infection Testing Among Substance-Using Sexual and Gender Minority Adolescents and Young Adults: Baseline Survey of a Randomized Controlled Trial
Source: JMIR Public Health Surveill. 2022 Jul 1;8(7):e30944. doi: 10.2196/30944 (PMC9288102; doi:10.2196/30944)
Supplement: Multimedia Appendix 3 [file publichealth_v8i7e30944_app3.pdf]

Multimedia Appendix 3. Distribution of demographic characteristics, structural factors, psychosocial barriers, and substance use and sexual behaviors by previous-year HIV and sexually transmitted infection (STI) testing among substance-using sexual and gender minority adolescents and young adults (N=414).

Characteristic	None (n=147)	STIs only (n=35)	HIV only (n=64)	Both (n=168)
**Demographics**				
Age (years), mean (SD)	21. 7 (3.15)	22.0 (3.6)	23.9 (3.2)	22.8 (3.0)
**Ethnicity, n (%)**				
Hispanic	17 (11.6)	3 (8.6)	4 (6.3)	16 (9.5)
Non-Hispanic	130 (88.4)	32 (91.4)	60 (93.8)	152 (90.5)
**Race, n (%)**				
White	110 (74.8)	23 (65.7)	35 (54.7)	117 (69.6)
Black/African American	11 (7.5)	8 (22.9)	13 (20.3)	24 (14.3)
Multiracial	13 (8.8)	3 (8.6)	8 (12.5)	13 (7.7)
Other	13 (8.8)	1 (2.9)	8 (12.5)	14 (8.3)
**Gender identity, n (%)**				
Cisgender men	113 (76.9)	23 (65.7)	61 (95.3)	134 (79.8)
Transgender men	23 (15.6)	8 (22.9)	0 (0)	15 (8.9)
Transgender women	5 (3.4)	2 (5.7)	2 (3.1)	4 (2.4)
Nonbinary	6 (4.1)	2 (5.7)	1 (1.6)	15 (8.9)
**Sexual identity, n (%)**				
Gay	90 (61.2)	17 (48.6)	49 (76.6)	114 (67.9)
Bisexual	35 (23.8)	9 (25.7)	10 (15.6)	23 (13.7)
Other	22 (15)	9 (25.7)	5 (7.8)	31 (18.5)
**Education, n (%)**				
Some high school	12 (8.2)	5 (14.3)	2 (3.1)	7 (4.2)
High school graduate/GED[a]	42 (28.6)	6 (17.1)	16 (25)	29 (17.3)
Some college or higher	93 (63.3)	24 (68.6)	46 (71.9)	132 (78.6)

**Employment, n (%)**				
Employed full-time	50 (34)	12 (34.3)	32 (50)	63 (37.5)
Other	97 (66)	23 (65.7)	32 (50)	105 (62.5)
**Housing, n (%)**				
Stable or permanent	94 (63.9)	25 (71.4)	41 (64.1)	101 (60.1)
Temporary, unstable, homeless, or other	53 (36.1)	10 (28.6)	23 (35.9)	67 (39.9)
**Yearly income (US $), n (%)**				
~14,999	50 (34.0)	20 (57.1)	18 (28.1)	69 (41.1)
15,000 to 39,999	46 (31.3)	7 (20.0)	24 (37.5)	52 (31.0)
~40,000	32 (21.8)	5 (14.3)	14 (21.9)	31 (18.5)
**Disability, n (%)**				
Yes	20 (13.6)	7 (20)	9 (14.1)	32 (19)
No	127 (86.4)	27 (77.1)	55 (85.9)	136 (81)
**Health insurance—current, n (%)**				
Yes	121 (82.3)	30 (85.7)	53 (82.8)	143 (85.1)
No	26 (17.7)	5 (14.3)	11 (17.2)	25 (14.9)
**Incarceration, n (%)**				
Never	115 (78.2)	32 (91.4)	49 (76.6)	133 (79.2)
Incarcerated in their lifetime but not incarcerated in the last 12 months	26 (17.7)	2 (5.7)	6 (9.4)	31 (18.5)
Incarcerated in the last 12 months	6 (4.1)	1 (2.9)	9 (14.1)	4 (2.4)
**HIV-related characteristics, n (%)**				
**Likelihood of HIV infection in the future**				
Very likely	1 (0.7)	0 (0)	0 (0)	2 (1.2)
Somewhat likely	13 (8.8)	4 (11.4)	5 (7.8)	18 (10.7)

Somewhat unlikely	61 (41.5)	17 (48.6)	25 (39.1)	67 (39.9)
Very unlikely	72 (49)	14 (40)	34 (53.1)	81 (48.2)
**Likelihood of HIV infection in the next 10 years**				
Very likely	3 (2)	3 (8.6)	0 (0)	11 (6.5)
Somewhat likely	25 (17)	8 (22.9)	12 (18.8)	29 (17.3)
Somewhat unlikely	65 (44.2)	15 (42.9)	27 (42.2)	54 (32.1)
Very unlikely	54 (36.7)	9 (25.7)	25 (39.1)	74 (44)
**PrEP[b] continuum**				
Unaware/aware	147 (100)	29 (82.9)	59 (92.2)	120 (71.4)
Past use	0 (0)	3 (8.6)	3 (4.7)	20 (11.9)
Current use	0 (0)	2 (5.7)	2 (3.1)	28 (16.7)
**Mental health, n (%)**				
**Anxiety—last 2 weeks**				
Minimal	43 (29.3)	6 (17.1)	20 (31.3)	51 (30.4)
Mild	41 (27.9)	9 (25.7)	21 (32.8)	48 (28.6)
Moderate	25 (17)	9 (25.7)	13 (20.3)	30 (17.9)
Severe	38 (25.9)	11 (31.4)	10 (15.6)	39 (23.2)
**Depression symptoms—previous week**				
Yes	94 (63.9)	23 (65.7)	35 (54.7)	100 (59.5)
No	52 (35.4)	11 (31.4)	29 (45.3)	68 (40.5)
**Substance use—previous 3 months, n (%)**				
**Tobacco use**				
Yes	125 (85)	30 (85.7)	50 (78.1)	133 (79.2)
No	22 (15)	5 (14.3)	14 (21.9)	35 (20.8)
**Hazardous drinking**				
Yes	47 (32)	9 (25.7)	25 (39.1)	66 (39.3)

No	100 (68)	26 (74.3)	39 (60.9)	102 (60.7)
Cannabis use	99 (67.3)	24 (68.6)	38 (59.4)	123 (73.2)
**Other drug use**	64 (43.5)	18 (51.4)	21 (32.8)	75 (44.6)
Stimulants	40 (27.2)	8 (22.9)	12 (18.8)	38 (22.6)
Sedatives	22 (15)	4 (11.4)	6 (9.4)	18 (10.7)
Club drugs	11 (7.5)	3 (8.6)	4 (6.3)	18 (10.7)
Opioids	12 (8.2)	0 (0)	4 (6.3)	4 (2.4)
Hallucinogens	25 (17)	3 (8.6)	6 (9.4)	24 (14.3)
Amyl-nitrites	17 (11.6)	6 (17.1)	8 (12.5)	34 (20.2)
**Sexual risk behavior—previous 3 months, n (%)**				
**CAI**[c]	71 (48.3)	22 (62.9)	43 (67.2)	21 (12.5)
Receptive CAI	59 (40.1)	19 (54.3)	36 (56.3)	100 (59.5)
Insertive CAI	43 (29.3)	13 (37.1)	23 (35.9)	84 (50)
**CVI**[d]	29 (19.7)	7 (20)	2 (3.1)	20 (11.9)
Receptive CVI	15 (10.2)	4 (11.4)	0 (0)	13 (7.7)
Insertive CVI	15 (10.2)	3 (8.6)	2 (3.1)	8 (4.8)

[a]GED: General Educational Development.
[b]PrEP: pre-exposure prophylaxis.
[c]CAI: condomless anal intercourse.
[d]CVI: condomless vaginal intercourse.
