# Supplementary material for: HIV and Sexually Transmitted Infection Testing Among Substance-Using Sexual and Gender Minority Adolescents and Young Adults: Baseline Survey of a Randomized Controlled Trial
Source: JMIR Public Health Surveill. 2022 Jul 1;8(7):e30944. doi: 10.2196/30944 (PMC9288102; doi:10.2196/30944)
Supplement: Multimedia Appendix 4 [file publichealth_v8i7e30944_app4.pdf]

Multimedia Appendix 4. Odds of lifetime HIV and sexually transmitted infection (STI) testing by demographic characteristics, structural factors, psychosocial barriers, and substance use and sexual behaviors among substance-using sexual and gender minority adolescents and young adults (N=414).

Characteristic	None versus both (reference)		STIs only versus both (reference)		HIV only versus both (reference)	
	OR[a] (95% CI)	*P* value	OR (95% CI)	*P* value	OR (95% CI)	*P* value
**Demographics**						
Age (years)	*0.79 (0.72-0.86)*[b]	*<.001*	*0.82 (0.72-0.94)*	*.003*	1.04 (0.94-1.16)	.43
**Ethnicity**						
Hispanic	1.82 (0.86-3.86)	.12	2.24 (0.78-6.46)	.13	0.26 (0.03-1.95)	.19
Non-Hispanic	1.00	—[c]	1.00	—	1.00	—
**Race**						
White	1.14 (0.68-1.92)	.63	1.10 (0.49-2.47)	.82	0.89 (0.46-1.72)	.72
Non-White	1.00	—	1.00	—	1.00	—
**Gender identity**						
Cisgender	1.32 (0.68-2.57)	*.41*	*0.23 (0.10-0.50)*	*<.001*	2.39 (0.82-6.99)	.11
Transgender or nonbinary	1.00	—	1.00	—	1.00	—
**Sexual identity**						
Gay[d]	0.88 (0.52-1.48)	.63	*0.29 (0.13-0.64)*	*.002*	0.98 (0.49-1.95)	.95

		Bisexual[d]	1.55 (0.82-2.92)	.18	*4.24 (1.87-9.59)*	*<.001*	2.06 (0.96-4.45)	.07
		Other[d]	0.78 (0.39-1.56)	.49	1.21 (0.47-3.13)	.70	0.35 (0.10-1.17)	.09
	Education[e]		*0.46 (0.31-0.68)*	*<.001*	*0.49 (0.27-0.88)*	*.02*	0.65 (0.38-1.12)	.12
	**Employment**							
		Employed full-time	0.67 (0.40-1.15)	.15	0.70 (0.31-1.61)	.41	1.64 (0.86-3.13)	.14
		Other	1.00	—	1.00	—	1.00	—
	**Housing**							
		Stable or permanent	0.88 (0.53-1.46)	.62	0.90 (0.41-1.99)	.80	0.77 (0.40-1.48)	.43
		Temporary, unstable, experiencing homeless, or other	1.00	—	1.00	—	1.00	—
	Yearly income[e]		1.07 (0.76-1.50)	.71	*0.52 (0.28-0.96)*	*.04*	1.16 (0.75-1.79)	.51
	**Disability**							
		Yes	0.63 (0.30-1.32)	.22	0.74 (0.25-2.22)	.59	1.05 (0.46-2.42)	.90
		No	1.00	—	1.00	—	1.00	—
	**Health insurance—current**							
		Yes	1.16 (0.57-2.38)	.69	0.68 (0.26-1.78)	.43	0.52 (0.24-1.11)	.09
		No	1.00	—	1.00	—	1.00	—

Incarceration[e]	1.16 (0.73-1.85)	.53	1.20 (0.60-2.42)	.60	1.67 (0.99-2.81)	.05
**HIV-related characteristics**						
Likelihood of HIV infection in the future[e]	0.92 (0.65-1.31)	.64	1.42 (0.77-2.62)	.26	1.12 (0.69-1.81)	.64
Likelihood of HIV infection in the next 10 years[e]	1.15 (0.85-1.54)	.37	1.11 (0.70-1.76)	.65	1.13 (0.77-1.67)	.53
PrEP[f] continuum[e]	—	—	0.33 (0.10-1.08)	.07	*0.25 (0.07-0.83)*	*.02*
**Mental health**						
Anxiety—last 2 weeks[e]	1.01 (0.82-1.26)	.90	*1.42 (1.01-1.99)*	*.047*	0.93 (0.70-1.25)	.64
**Depression symptoms—previous week**						
Yes	1.35 (0.80-2.26)	.26	2.19 (0.90-5.32)	.08	0.88 (0.46-1.69)	.70
No	1.00	—	1.00	—	1.00	—
**Substance use—previous 3 months**						
**Tobacco use**						
Yes	1.33 (0.70-2.54)	.39	3.64 (0.84-15.75)	.08	2.05 (0.77-5.45)	.15
No	1.00	—	1.00	—	1.00	—
**Hazardous drinking**						
Yes	0.74 (0.44-1.25)	.26	0.62 (0.26-1.44)	.27	0.78 (0.39-1.55)	.48
No	1.00	—	1.00	—	1.00	—

Cannabis use[g]	1.06 (0.62-1.81)	.84	1.43 (0.59-3.48)	.43	0.70 (0.36-1.35)	.29
**Other drug use[g]**	0.99 (0.60-1.63)	.97	0.91 (0.42-1.99)	.82	0.85 (0.44-1.63)	.62
Stimulants[g]	1.08 (0.60-1.92)	.80	1.53 (0.66-3.53)	.32	1.03 (0.48-2.21)	.95
Sedatives[g]	*2.05 (1.04-4.03)*	*.04*	0.64 (0.14-2.83)	.55	1.13 (0.41-3.12)	.81
Club drugs[g]	0.95 (0.39-2.29)	.90	0.76 (0.17-3.40)	.72	1.05 (0.35-3.21)	.93
Opioids[g]	*4.38 (1.58-12.16)*	*.005*	1.29 (0.15-10.83)	.82	2.70 (0.67-10.87)	.16
Hallucinogens[g]	1.89 (0.99-3.64)	.06	1.53 (0.55-4.31)	.42	0.97 (0.35-2.64)	.95
Amyl-nitrites[g]	0.54 (0.25-1.14)	.11	0.33 (0.08-1.42)	.14	0.71 (0.29-1.79)	.47
**Sexual risk behavior—previous 3 months**						
**CAI[h,i]**	*0.35 (0.21-0.58)*	*<.001*	*0.26 (0.12-0.58)*	*.001*	0.80 (0.41-1.59)	.53
Receptive CAI[i]	*0.50 (0.30-0.82)*	*.006*	*0.27 (0.12-0.64)*	*.003*	0.68 (0.36-1.30)	.25
Insertive CAI[i]	1.89 (0.69-5.22)	.22	0.73 (0.28-1.91)	.52	2.23 (0.95-5.22)	.06
**CVI[j,i]**	1.20 (0.59-2.45)	.62	*3.19 (1.34-7.62)*	*.009*	0.93 (0.34-2.55)	.89

		Receptive CVI[i]	0.64 (0.21-1.94)	.43	*3.30 (1.20-9.07)*	*.02*	0.95 (0.27-3.35)	.93
		Insertive CVI[i]	1.74 (0.71-4.25)	.23	1.88 (0.51-6.91)	.34	0.79 (0.18-3.60)	.76

[a]OR: odds ratio.

[b]P<.05.

[c] Reference (not applicable).

[d]The odds for selected sexual identity divided by the odds for other sexual identity.

[e]Ordinal variables were considered as continuous variables in the modeling; education was ordered as (1) some high school, (2) high school graduate/General Educational Development, and (3) some college or higher; income was ordered as (1) ~US $14,999, (2) US $15,000 to US $39,999, and (3) ~US $40,000; incarceration was ordered as (1) never, (2) incarcerated in their lifetime but not incarcerated in the last 12 months, and (3) incarcerated in the last 12 months; likelihood of HIV infection was ordered as (1) very likely, (2) somewhat likely, (3) somewhat unlikely, and (4) very unlikely; pre-exposure prophylaxis continuum was ordered as (1) unaware/aware, (2) previous use, and (3) current use; and anxiety was ordered as (1) minimal, (2) mild, (3) moderate, and (4) severe.

[f]PrEP: pre-exposure prophylaxis.

[g]The odds for drug use divided by the odds for no drug use.

[h]CAI: condomless anal intercourse.

[i]The odds for condomless intercourse divided by the odds for no condomless intercourse. OR=1.00 indicates the reference group.

[j]CVI: condomless vaginal intercourse.
