# Supplementary material for: HIV and Sexually Transmitted Infection Testing Among Substance-Using Sexual and Gender Minority Adolescents and Young Adults: Baseline Survey of a Randomized Controlled Trial
Source: JMIR Public Health Surveill. 2022 Jul 1;8(7):e30944. doi: 10.2196/30944 (PMC9288102; doi:10.2196/30944)
Supplement: Multimedia Appendix 5 [file publichealth_v8i7e30944_app5.pdf]

Multimedia Appendix 5. Odds of previous-year HIV and sexually transmitted infection (STI) testing by demographic characteristics, structural factors, psychosocial barriers, and substance use and sexual behaviors among substance-using sexual and gender minority adolescents and young adults (N=414).

Characteristic	None versus both (reference)		STIs only versus both (reference)		HIV only versus both (reference)	
	OR[a] (95% CI)	*P* value	OR (95% CI)	*P* value	OR (95% CI)	*P* value
**Demographics**						
Age (years)	*0.89 (0.83-0.96)*[b]	*.002*	0.93 (0.82-1.04)	.19	*1.12 (1.02-1.23)*	*.02*
**Ethnicity**						
Hispanic	1.24 (0.60-2.56)	.56	0.89 (0.25-3.24)	.86	0.63 (0.20-1.97)	.43
Non-Hispanic	1.00	—[c]	1.00	—	1.00	—
**Race**						
White	1.26 (0.79-2.02)	.34	0.74 (0.35-1.55)	.43	0.63 (0.35-1.13)	.12
Non-White	1.00	—	1.00	—	1.00	—
**Gender identity**						
Cisgender	0.84 (0.49-1.144)	.53	0.49 (0.22-1.08)	.08	*5.16 (1.53-17.45)*	*.008*
Transgender or nonbinary	1.00	—	1.00	—	1.00	—
**Sexual identity**						
Gay[d]	0.75 (0.47-1.19)	.22	*0.45 (0.21-0.94)*	*.03*	1.55 (0.80-3.00)	.20

Bisexual[d]	*1.97 (1.10-3.52)*	*.02*	2.18 (0.91-5.24)	.08	1.17 (0.52-2.61)	.71
Other[d]	0.78 (0.43-1.41)	.41	1.53 (0.65-3.59)	.33	0.38 (0.14-1.01)	.05
Education[e]	*0.57 (0.39-0.84)*	*.005*	0.56 (0.31-1.00)	.05	0.82 (0.49-1.39)	.47
**Employment**						
Employed full-time	0.86 (0.54-1.36)	.52	0.87 (0.41-1.87)	.72	1.67 (0.93-2.98)	.09
Other	1.00	—	1.00	—	1.00	—
**Housing**						
Stable or permanent	1.18 (0.75-1.86)	.49	1.66 (0.75-3.67)	.02	1.18 (0.65-2.15)	.58
Temporary, unstable, homeless, or other	1.00	—	1.00	—	1.00	—
Yearly income[e]	1.20 (0.89-1.62)	.24	0.67 (0.39-1.15)	.14	1.34 (0.91-1.98)	.14
**Disability**						
Yes	0.67 (0.36-1.23)	.20	1.10 (0.44-2.75)	.84	0.70 (0.31-1.55)	.38
No	1.00	—	1.00	—	1.00	—
**Health insurance—current**						
Yes	0.81 (0.45-1.48)	.50	1.05 (0.37-2.96)	.93	0.84 (0.39-1.83)	.67
No	1.00	—	1.00	—	1.00	—

Incarceration[e]	1.10 (0.72-1.68)	.66	0.53 (0.20-1.41)	.21	1.54 (0.95-2.51)	.08
**HIV-related characteristics**						
Likelihood of HIV infection in the future[e]	1.08 (0.78-1.49)	.64	0.88 (0.53-1.46)	.61	1.25 (0.81-1.93)	.31
Likelihood of HIV infection in the next 10 years[e]	1.03 (0.79-1.34)	.84	0.70 (0.46-1.05)	.08	1.10 (0.78-1.56)	.59
PrEP[f] continuum[e]	—	—	0.66 (0.37-1.19)	.16	*0.36 (0.19-0.70)*	*.003*
**Mental health**						
Anxiety—last 2 weeks[e]	1.04 (0.86-1.27)	.67	1.34 (0.97-1.85)	.08	0.90 (0.69-1.16)	.41
**Depression symptoms—previous week**						
Yes	1.23 (0.78-1.94)	.38	1.42 (0.65-3.11)	.38	0.82 (0.46-1.47)	.51
No	1.00	—	1.00	—	1.00	—
**Substance use—previous 3 months**						
**Tobacco use**						
Yes	1.50 (0.83-2.69)	.18	1.58 (0.57-4.37)	.38	0.94 (0.47-1.89)	.86
No	1.00	—	1.00	—	1.00	—
**Hazardous drinking**						
Yes	0.73 (0.46-1.16)	.18	0.54 (0.24-1.21)	.14	0.99 (0.55-1.79)	.98
No	1.00	—	1.00	—	1.00	—

Cannabis use[g]	0.76 (0.46-1.23)	.26	0.80 (0.36-1.76)	.58	*0.54 (0.29-0.98)*	*.04*
**Other drug use[g]**	0.96 (0.61-1.49)	.84	1.31 (0.63-2.72)	.46	0.61 (0.33-1.11)	.10
Stimulants[g]	1.28 (0.77-2.14)	.35	1.01 (0.43-2.41)	.98	0.79 (0.38-1.63)	.52
Sedatives[g]	1.47 (0.75-2.86)	.26	1.08 (0.34-3.40)	.90	0.86 (0.33-2.28)	.77
Club drugs[g]	0.67 (0.31-1.48)	.33	0.78 (0.22-2.81)	.71	0.56 (0.18-1.71)	.31
Opioids[g]	*3.64 (1.15-11.56)*	*.03*	—	—	2.73 (0.66-11.28)	.16
Hallucinogens[g]	1.23 (0.67-2.26)	.51	0.56 (0.16-1.98)	.37	0.62 (0.24-1.60)	.32
Amyl-nitrites[g]	*0.52 (0.27-0.97)*	*.04*	0.82 (0.31-2.12)	.68	0.56 (0.25-1.29)	.18
**Sexual risk behavior—previous 3 months**						
**CAI[h,i]**	*0.36 (0.23-0.58)*	*<.001*	0.66 (0.31-1.41)	.28	0.80 (0.43-1.48)	.47
Receptive CAI[i]	*0.46 (0.29-0.72)*	*<.001*	0.81 (0.39-1.68)	.57	0.87 (0.49-1.57)	.65
Insertive CAI[i]	0.95 (0.40-2.23)	.91	0.70 (0.32-1.51)	.37	1.69 (0.80-3.58)	.17
**CVI[j,i]**	1.82 (0.98-3.38)	.06	1.85 (0.72-4.79)	.21	0.24 (0.05-1.05)	.06

		Receptive CVI[i]	1.36 (0.62-2.95)	.44	1.54 (0.47-5.03)	.48	—	—
		Insertive CVI[i]	2.27 (0.94-5.53)	.07	1.88 (0.47-7.45)	.37	0.65 (0.13-3.12)	.59

[a]OR: odds ratio.

[b]P<.05.

[c] Reference (not applicable).

[d]The odds for selected sexual identity divided by the odds for other sexual identity.

[e]Ordinal variables were considered as continuous variables in the modeling; education was rated (1) some high school, (2) high school graduate/General Educational Development, and (3) some college or higher; income was ordered as (1) ~US $14,999, (2) US $15,000 to US $39,999, and (3) ~$40,000; incarceration was ordered as (1) never, (2) incarcerated in their lifetime but not incarcerated in the last 12 months, and (3) incarcerated in the last 12 months; likelihood of HIV infection was ordered as (1) very likely, (2) somewhat likely, (3) somewhat unlikely, and (4) very unlikely; pre-exposure prophylaxis continuum was ordered as (1) unaware/aware, (2) past use, and (3) current use; and anxiety was ordered as (1) minimal, (2) mild, (3) moderate, and (4) severe.

[f]PrEP: pre-exposure prophylaxis.

[g]The odds for drug use divided by the odds for no drug use.

[h]CAI: condomless anal intercourse.

[i]The odds for condomless intercourse divided by the odds for no condomless intercourse. OR=1.00 indicates the reference group.

[j]CVI: condomless vaginal intercourse.
